# Interventions That Successfully Reduced Adults Salt Intake—A Systematic Review

**DOI:** 10.3390/nu14010006

**Published:** 2021-12-21

**Authors:** Tânia Silva-Santos, Pedro Moreira, Micaela Rodrigues, Patrícia Padrão, Olívia Pinho, Pedro Norton, Altin Ndrio, Carla Gonçalves

**Affiliations:** 1 Faculty of Nutrition and Food Sciences, University of Porto, 4150-180 Porto, Portugal; pedromoreira@fcna.up.pt (P.M.); micaela.rodrigues97@hotmail.com (M.R.); patriciapadrao@fcna.up.pt (P.P.); oliviapinho@fcna.up.pt (O.P.); carlagoncalves.pt@gmail.com (C.G.); 2 EPIUnit—Institute of Public Health, University of Porto, 4200-450 Porto, Portugal; pedrosnorton@gmail.com; 3 CIAFEL—Research Centre in Physical Activity, Health and Leisure, Faculty of Sport, University of Porto, 4200-450 Porto, Portugal; 4 LAQV-REQUIMTE—Laboratory of Bromatology and Hydrology, Faculty of Pharmacy, University of Porto, 5000-801 Porto, Portugal; 5 Occupational Health Service of Centro Hospitalar, Universitário de São João, E.P.E., 4200-319 Porto, Portugal; 6 Clinical Pathology Service of Centro Hospitalar, Universitário de São João, E.P.E., 4200-319 Porto, Portugal; altin.ndrio@chsj.min-saude.pt; 7 CITAB—Centre for the Research and Technology of Agro-Environmental and Biological Sciences, University of Trás-os-Montes and Alto Douro, 5000-801 Vila Real, Portugal

**Keywords:** salt reduction, sodium, behavior change, hypertension, dietary intervention

## Abstract

Background: Adequate sodium intake is important for lowering blood pressure and thus reducing cardiovascular disease risk and other complications. The aim of this review is to identify recent interventions around the world that have been successful in reducing salt intake. Methods: A search in the PubMed, Web of Science and Scopus databases was performed. We include studies published in the last 10 years; randomized trials, pilot intervention without a control arm or experimental study; adult participants; and interventions that successfully reduced salt intake. Study quality was assessed. Results: We included 21 studies, 16 randomized intervention trials and five nonrandomized intervention studies. Eleven interventions described health and nutritional education, seven interventions described nutritional education plus other interventions, and three studies used salt meters to reduce sodium intake. Conclusion: Health and nutritional education, nutritional education plus other interventions and estimates of salt intake showed success in the reduction of salt consumption. There is no evidence that one type of intervention analyzed is more effective than other in reducing salt consumption, so we must analyze each in which individuals or subpopulations will have the intervention performed and use the most suitable approaches to lead to better results.

## 1. Introduction

Noncommunicable diseases are the main factor for global morbidity and mortality. Approximately 17 million people die annually from cardiovascular diseases and about 9.4 million of these deaths are due to complications of hypertension [1,2].

Excessive sodium in the diet increases blood pressure and therefore increases the risk of cardiovascular diseases [3]. It is estimated that 3 million deaths worldwide are associated with high sodium intake [4].

Reducing sodium intake is important to lower blood pressure and thus reduce cardiovascular diseases and other complications associated with high sodium intake, such as chronic kidney disease, obesity, gastric cancer and liver diseases. The most common form of sodium consumption is sodium chloride, commonly known as table salt [5,6]. Reducing salt intake by 3 g per day is projected to reduce the annual number of new coronary heart disease cases by 60,000 to 120,000, stroke by 32,000 to 66,000, myocardial infarction by 54,000 to 99,000, and myocardial infarction by 44,000 to 92,000 the annual number of deaths from any cause. It would save 194,000 to 392,000 quality-adjusted life years and $10 billion to $24 billion in health care costs annually [7].

The World Health Organization (WHO) has flagged population salt reduction as one of the five priority interventions to prevent noncommunicable diseases. The WHO has adopted a global target of 30% reduction in the mean salt intake by the population until 2025 [8,9].

In 2014, 75 countries with national salt reduction strategies were identified, more than double the 32 reported in 2010. However, there are limited examples of effective strategies to reduce dietary salt intake around the world and uncertainty about the specific initiatives or elements of the strategy that are central to its success [10,11].

In 2016, WHO published the SHAKE package to assist in the development, implementation and monitoring of salt reduction by the population, based on five principles; namely surveillance, harness industry, adopt standards for labeling and marketing, knowledge and environment [12]. In countries where salt added to the table or during cooking is the main source of salt intake, education and communication strategies are important to influence the behavior of consumers, cooks and suppliers to reduce the use of salt. Educational interventions provide consumers with information, education or skills to reduce salt intake, altering people’s salt behavior, strengthening knowledge of salt and its adverse effects and abilities to help reduce salt intake [11,12]. In countries where processed foods are the main source of salt, the food industry and government policy makers are the target audience. However, consumer engagement gained through education and communication can put pressure on the food industry to follow through on salt reduction commitments [12].

Previous reviews have evaluated interventions to reduce salt intake [13,14]. Our review summarizes recent interventions that have been successful in reducing salt as measured by urinary excretion measures. Therefore, the aim of this review is to identify interventions around the world over the past ten years that have been successful in reducing salt intake.

## 2. Materials and Methods

The systematic review followed the recommendations of the Cochrane collaboration method [15] and was written according to the Preferred Reporting Items for Systematic Reviews and Meta-Analyzes (PRISMA) [16]. The review protocol was registered with the International prospective register of systematic reviews (PROSPERO), the University of York Center for Reviews and Dissemination (CRD42020221165).

### 2.1. Eligibility Criteria

#### 2.1.1. Types of Studies

We include studies published in the last 10 years and written in English, Portuguese, French or Spanish. Randomized studies, pilot interventions without a control arm and experimental studies were included in this review.

#### 2.1.2. Types of Participants

Studies of all populations, adults (>18 years) and living in any region worldwide. We excluded studies that not exclusively targeted on sodium intake, but on various health behaviors (e.g., physical activity) and participants with kidney disease.

#### 2.1.3. Types of Intervention

This review focused on interventions that successfully reduced salt intake. Success in reducing salt was defined as a statistically significant reduction (*p* < 0.05). Studies with an intervention period of less than four weeks were excluded.

#### 2.1.4. Types of Outcome Measures

The primary outcome of this review was the reduction in salt intake estimated by urinary measurements (24-h urine collection and urine spot). The secondary outcome was changes in blood pressure values.

### 2.2. Information Sources

The studies were identified by searching electronic databases and scanning reference lists of the articles included in this article and in systematic reviews that emerged in the search for data. The databases searched were PubMed, Web of Science and Scopus. The research period in November 2020 was the last 10 years (between 2010 and October 2020); however, processing the review data took time and to ensure that the evidence was current and applicable to the current environment, additional research was carried out to include the period between October 2010 and August 2021, but no additional articles were found that met the inclusion criteria.

### 2.3. Search Strategy

The search was performed by one author (TS-S) and included two categories: “Interventions for reducing salt intake” terms and “Urinary measures” terms.

The search terms used in PubMed were as follows: (Intervention or “Food programmes” or “food policy” or “meal plan” or “food and nutrition education” or “health promotion” or activity or project or campaign or initiative or marketing or media) and ((“dietary salt” or “dietary sodium”) or ((salt OR sodium) and (consumption or intake ordiet orfood ornutrition ordietetics))) and (urin*). These terms have been adapted for research on the Web of Science and Scopus.

### 2.4. Selection Process

Two authors (TS-S, MR) independently selected the titles and abstracts first and then the full text of the studies. Discrepancies in the selections were discussed until consensus was reached. When no agreement was reached, a third author decided (CG).

### 2.5. Data Collection Process

Two authors (TS-S, MR) independently extracted all data and verified the extracted data. Disagreements were resolved by discussion between the two.

Data were extracted according to the Cochrane handbook for systematic intervention reviews [15] and included author, publication year, title, country, participants (e.g., number, age, sex), study eligibility criteria, study design, recruitment and sampling procedures, enrolment start and end dates; length of participant follow-up, random sequence generation, allocation sequence concealment, masking for randomized trials, and methods used to prevent and control for confounding, selection biases, and information biases for non-randomized studies, methods used to prevent and address missing data, statistical analysis, source(s) of funding or/and potential conflicts of interest, description of the intervention(s) and comparison intervention(s), type of urinary measurements, laboratory method used to analyze urine, method of aggregation (e.g., mean and standard deviation of sodium intake and blood pressure values before and after the intervention), number of urine collections, timing of outcome measurements, number of participants randomly assigned and included in the analysis; and number of participants who withdrew, were lost to follow-up or were excluded (with reasons for each).

### 2.6. Study Risk of Bias Assessment

The quality of the study was independently assessed by two reviewers (TS-S, MR). Discrepancies in the selections were discussed until consensus was reached. The risk of bias in randomized clinical trials was assessed using the Cochrane risk of bias tool (RoB 2) [17] and in non-randomized intervention studies using the ROBINS-I tool [18].

RoB tool considered the assessment of bias in five domains; namely, randomization process, deviations from intended interventions, missing results data, measurement of the outcome and selection of reported results. The judgments of risk of bias in each domain are assessed as low risk of bias, some concerns or high risk of bias. According to each domain assignment, an overall risk of bias judgment was made.

The ROBINS-I tool includes seven domains divided by pre-intervention and at-intervention (confounding, selection of participants into the study, classification of intervention) and post-intervention (derivations from intended intervention, missing data, measurement of outcomes, selection of the reported result). The results of the judgment for each domain and for the final overall bias were categorized as low, moderate, serious, or critical risk of bias, or no information.

## 3. Results

### 3.1. Study Selection

**Figure 1:**
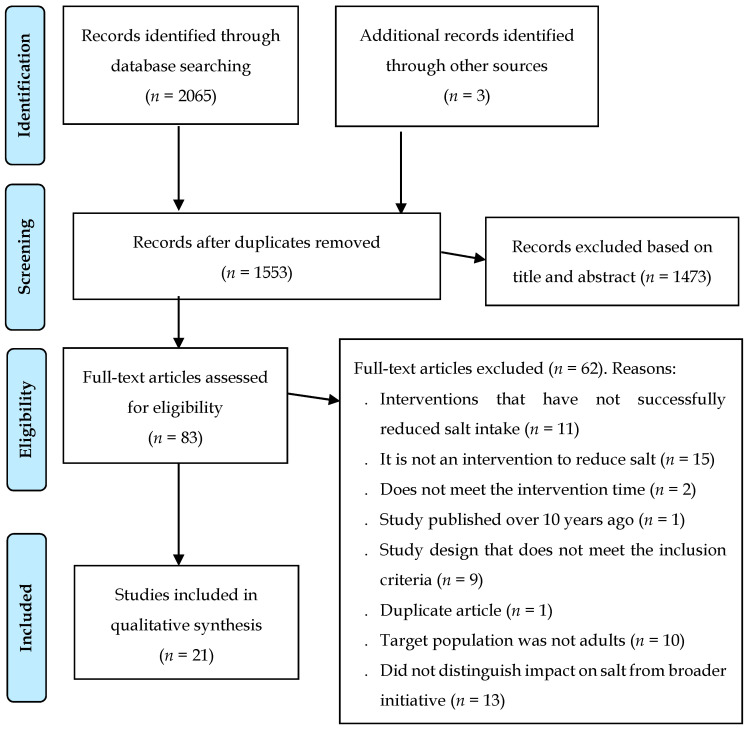
PRISMA flow chart of included studies.

The search identified 2065 records and three additional records were added by scanning the reference lists of articles included in this article and in systematic reviews that emerged from the data search. After discarding duplicates, 1553 abstracts were selected to review titles and abstracts, leaving 83 complete articles to be evaluated for eligibility. Of these, 62 full articles were excluded; the reasons for the exclusion of these studies were: 14 studies in which the interventions did not successfully reduce salt intake, 15 were not an intervention to reduce salt, two did not meet the intervention time, one study was published over 10 years ago, nine studies with study design did not meet the inclusion criteria, one was a duplicate article, there were 10 studies in which the target population was not adult, and 10 studies did not distinguish the impact on salt consumption of a broader initiative. After screening, 21 articles were included for qualitative synthesis (Figure 1).

### 3.2. Study Characteristics

Table 1 and Table 2 describe an overview of all included studies.

#### 3.2.1. Participants Characteristics

All studies were published between 2010 and 2020; the sample of each study ranged between 30 and 753 participants; the median of participants was 117. Eleven studies were conducted in Asia (Japan [19,20,21,22,23], Iran [24,25,26], China [27,28] and Thailand [29]), four studies were conducted in Europe (Republic of Ireland [30], Denmark [31], Bosnia-Herzegovina [32] and Italy [33]), four studies conducted in America (United States [34,35,36] and Brazil [37]) and two studies conducted in Australia [38,39].

Eleven studies were conducted in the general population [19,20,23,25,26,27,28,31,36,38,39], eight studies in participants with hypertension or pre-hypertension [21,22,24,30,32,33,34,37], one in people at high risk of cardiovascular disease [29], and one study in heart failure participants [35].

Participants in the studies included in this review were recruited from the community (*n =* 10) [19,23,25,26,28,30,31,34,38,39], clinics for the treatment of hypertension, health centers or hospitals (*n =* 5) [21,29,32,35,37], a railway company (*n =* 1) [22], from other previous study (*n =* 1) [24], in a school (*n =* 1) [27], in a university (*n =* 1) [36] and in several locations, including an agricultural cooperative, a hospital, and two cities (*n =* 1) [20]. One study included in this review did not contain information about the place of recruitment the participants [33].

#### 3.2.2. Interventions Characteristics

The present work included 16 randomized intervention trials [19,20,21,22,24,27,28,29,30,31,32,34,35,36,37,38] and five nonrandomized intervention studies [23,25,26,33,39]. All used a pre- and post-test design, except for one study [28], which used only a post-test design.

Most studies (*n =* 15) compared a group of intervention participants with a control group of participants, two studies compared two different interventions [36,38], two studies compared a control group with two interventions groups [31,35], and two studies had no control group [23,39]. The intervention period ranged between four weeks and 18 months.

Must studies have described education interventions to reduce salt intake. Seven interventions were just health and nutritional education [21,24,25,27,33,35,38], eleven interventions were nutritional education plus other interventions [19,22,26,28,29,30,31,32,34,37,39] and three studies used only salt meters to reduce sodium intake, an app and a urine sodium meter [20,23,36].

#### 3.2.3. Outcome Characteristics

Salt consumption was assessed by urinary sodium excretion, as defined in the methodology of this review. The included studies used different methods, and most used 24-h urinary excretion (*n =* 13) [21,24,27,28,30,31,32,33,34,35,37,38,39], followed by spot urine collection (*n =* 5) [19,20,25,26,36] and overnight urine sample (*n =* 3) [22,23,29]. The number of urine collections in the studies that used 24-h urinary excretion to assess salt intake varied: one collection twice (*n =* 5) [21,24,33,37,39], one collection at three times (*n **=*** 4) [30,32,35,38], three consecutive collections in twice (*n =* 1) [31], two collections consecutive at two times (*n =* 1) [27], only one collection at the end of the study (*n =* 1) [28] and four times of urine collections, in which three times they asked to collect two days followed and at one time only one urine collection (*n =* 1) [34]. In the studies that used the urinary spot to assess salt consumption, four studies requested a collection in two moments [19,20,25,26] and one study requested two collections also in two moments [36]. The studies that measured salt through overnight urinary excretion used the following methodology: urine collection for three consecutive nights at three different times (*n =* 1) [29], urine collection during the first week of intervention and in the last week (*n =* 1) [22], urine collection for four weeks (*n =* 1) [23].

Fifteen studies assessed participants’ blood pressure [19,20,21,22,23,24,26,27,28,29,33,36,37]. Most studies assessed blood pressure twice (*n =* 12) and only three assessed blood pressure three times [29,30,32].

### 3.3. Types of Interventions

The interventions were summarized and categorized into: (1) Health and nutritional education; (2) Nutritional education plus other interventions; and (3) Estimates of salt intake.

All studies included in the review showed statistically significant differences in salt reduction between the intervention group and the control group. With the exception of the two studies [23,39] that did not have a control group, success in reducing salt was verified between the baseline period and after the intervention.

#### 3.3.1. Health and Nutritional Education

Interventions in health and nutrition education were carried out mainly by health professionals (nutritionists, doctors, psychologists and nurses) [21,24,25,33,35,38] with the exception of one intervention, carried out by health educators trained by researchers [27].

Ireland et al. [38] described that dietary education was provided in groups of four to five in 15-min sessions, in which the participants were informed that the purpose of the study was to reduce salt intake and were instructed to continue their usual dietary patterns using either the Tick symbol or the Food Standards Australia New Zealand guideline to identify reduced salt foods. In addition, participants in both groups were provided with a list of low-sodium foods and a second 10-min one-on-one session in week 4 [38].

Nakano et al. [21] described that they provided participants with intensive nutritional education held five times during the intervention period, lasting 20 min. At the time of the first and fifth sessions of nutrition education, each patient answered the Food Frequency Questionnaire and performed a survey to determine the amount of salt intake. Nutrition education sessions were customized based on individual questionnaire and survey results [21]. Also, Rahimde et al. [24] based on the results of the pre-test, developed educational content in the form of a booklet containing information about salt and its consumption rate in Iran, the definition of blood pressure, the effects of high salt intake, sources of salt intake and diseases associated, salty foods, ways to reduce salt intake and the amount of salt in different foods. The educational intervention included 10 educational sessions using slides and a blackboard, and during the sessions, booklets were distributed among the participants. The authors defined the intervention as an education program based on the theory of planned behavior for salt intake [24].

In the study by Layeghias et al. [25] described that the intervention group received an educational package with the aim of reducing salt consumption and using alternatives, through posters for installation in the kitchen, leaflets, free telephone service, four educational classes and brief interventions by doctors and other health professionals [25].

Dunbar et al. [35] described a three-group intervention (usual care, patient family education, and family partnership intervention). In the usual care group, they only provided usual care and educational pamphlets created by the Heart Failure Society of America. In the patient’s family education group, they provided the usual care, pamphlets and added educational content written and on DVD, a second group session with their family member, received feedback on their usual sodium intake and after 4 months they received a telephone education reinforcement session. In the family partnership intervention group, participants received the same education as the other two groups, plus 2 group sessions that focused on teaching family members how to support, communicate, empathize and empower each other’s roles. They also received written information about family partnership and support for autonomy [35].

The intervention described by Musso et al. [33] was a low-sodium diet prescribed by the nutritionist. The diet was based on simple recommendations printed on a single sheet of A4 paper.

He et al. [27] developed an educational program in which program materials were developed around cartoon characters and consisted of lesson plans, activity worksheets, and homework assignments. In the intervention group, the usual health education classes were replaced by classes on salt reduction. Classes included lectures with the participation of the family and posters were also placed in the classroom about the harmful effects of salt and how to reduce salt consumption. The children were instructed to emphasize the 50% salt reduction goal at home and to remind the whole family of this goal after each lesson, to deliver salt reduction messages, salt reduction methods and skillful tips for the whole family and to develop a salt reduction action plan for their own family and oversee actions at home. Parents received educational materials in the form of a newsletter that covered topics such as salt and its effects on blood pressure and cardiovascular disease, the main sources of dietary salt, and cooking with less salt. The researchers monitored the child’s family’s use of salt, verified how much the family’s salt use differed from the established goal, and communicated the results to them. Each family also received a control spoon of salt (2 g of salt) [27].

#### 3.3.2. Effects of Interventions

In the study by Ireland et al. [38] mean salt intake decreased by around 0.9 g/day (from 7.3 ± 3.0 g/day, *p* < 0.05) in the tick group and in the Food group Australia New Zealand standards mean salt intake decreased by around 2.0 g/day (from 7.9 ± 2.6 g/day, *p* < 0.05).

Nakano et al. [21] reported a decrease by around 1.8 g/salt per day (from 8.6 ± 3.2, *p* = 0.002) in the intervention group. Ambulatory 24-h systolic blood pressure was significantly lowered in the intervention group (−4.5 ± 1.3 mmHg) compared with the control group (2.8 ± 1.4 mm Hg), *p* < 0.001).

Rahimdel et al. [24] showed that the mean salt intake decreased by 4.7 g/day (from 12.9 ± 4.4 g/day, *p* < 0.001) in the intervention group. There were no differences in blood pressure.

In the study by Dunbar et al. [35] mean salt intake decreased by around 3.1 g/day (from 9.0 ± 4.4 g/day, *p* < 0.05) in the family partnership intervention.

He et al. [27] described that in adults, mean salt intake decreased by around 2.1 g/day (from 12.6 ± 0.4 g/day, *p* < 0.001) in the intervention group and the difference mean between groups was −2.9 (*p* < 0.001). The difference mean between groups on systolic blood pressure was −2.3 mm Hg, *p* < 0.05. The effect on diastolic blood pressure was not significant.

In the study by Musso et al. [33] mean salt intake decreased by around 1.1 g/day (from 8.8 ± 2.6 g/day, *p <* 0.05). Systolic and diastolic blood pressure in the intervention group also decreased (134.16 to 126.5 mmHg, *p* = 0.014 and 80.59 to 75.9 mmHg, *p* = 0.026, respectively).

Layeghiasl et al. [25] showed that mean salt intake decreased by 3.01 g/day in the intervention group (from 14.34 g/day, *p<* 0.001).

#### 3.3.3. Nutritional Education plus Other Interventions

Multicomponent educational interventions that, in addition to health and nutrition education, used salt substitutes (*n =* 2) [28,37], low-salt bread (*n =* 3) [26,30,31], urine-excreted salt meter (*n =* 1) [22], health campaigns large-scale awareness (*n =* 1) [39], cooking classes (*n =* 1) [19], warning stickers (*n =* 1) [32], digital handheld pocket salt meter (*n =* 1) [29] and a sodium-specific tracking tool (*n =* 1) [34]. Interventions were delivered by health professionals (*n =* 5) [19,22,29,30,32], health educators (*n =* 1) [28], study counselors (*n =* 1) [34] and bakers (*n =* 1) [26]. Three studies did not discriminate who delivered the interventions [31,37,39].

Cashman et al. [30] described that during the intervention period they provided participants with pragmatic dietary advice, replacing bread and a limited number of other foods with equivalent foods with lower salt content. At the beginning of the salt restriction period, participants were given a list of foods that contained common salt (salty and naturally salty) and asked to limit their consumption of these foods. Subjects received brown or white sliced bread with low salt content (0.3 g/100 g), unsalted margarine and received lunch meats without added salt (turkey and cooked meat), if desired (optional) [30]. In the study by Riis et al. [31] they also provided bread with a lower sodium content, which was distributed free of charge twice a week. During the first 2 weeks of intervention, the sodium content in bread was similar to the average content in supermarket and bakery bread. In both intervention groups, the sodium content was gradually reduced by 0.08 g per 100 g (0.2 g salt/100 g) each week until the sodium content reached 0.24 g per 100 g (0.6 g salt/100 g) on rye bread and 0.16 g per 100 g (0.4 g salt/100 g) on wheat bread, which remained for the remainder of the intervention. In Intervention A, they reduced only the sodium content of the bread, but in Intervention B they combined it with a dietary counseling program. Diet counseling consisted of a 2-h group introduction, a 1-h family counseling session, followed by two telephone counseling sessions with a parent, and weekly emails [31]. In the study by Jafari et al. [26] reduced the salt content by 40% over 4 weeks. They gave lectures on the harmful effects of salt, posted banners in squares and crossroads, and posters on the harmful effects of salt in all bakeries and supermarkets. In selected homes, they distributed a leaflet about salt damage. The main intervention in the intervention municipality was the gradual reduction in the consumption of salt in people’s diets through bread [26].

Li et al. [28] described a salt reduction program that comprised community-based health education and availability to purchase added-potassium salt substitute from village stores. The health education component consisted of public lectures, exhibition and distribution of promotional materials, and special interactive education sessions aimed at individuals at high risk for vascular disease [28]. In the study by Barros et al. [37] all participants were instructed to consume only the salt provided throughout the study and to reduce their consumption of foods rich in sodium. They provided 28 plastic bags containing the daily amount of salt for each participant. The light salt consisted of 130 mg of sodium, 346 mg of potassium and 44 mcg of iodine per gram [37].

Morikawa et al. [22] described that in the first and last week of the study, participants in the intervention group measured daily urinary salt excretion using the electronic salt sensor. In addition, participants received an email 10 times during the study period with information about the salt content of foods, salt reduction methods and a message encouraging a salt-reduced diet [22].

The salt reduction intervention described by Land et al. [39] targeted the whole community and was based upon the Communication for Behavioral Impact (COMBI) framework. This framework utilizes an integrated communication model to enact community advocacy and impact, so there were several meetings held with local government, local doctors, health professionals, five of the largest employers, with the local business association, business owners (mostly owners of cafes, bars and restaurants) and community groups. The local communication channels were all directed with information and stories about the program. Information booths were installed in the two main commercial areas and around 500 individual houses were visited by two employees who worked in this activity. They used salt substitutes and the “FoodSwitch” smartphone app to encourage a reduction in salt intake. The salt substitute consisted of 136 mg of sodium and 176 mg of potassium per 0.8 g serving and was made available free of charge for use by consumers. The smartphone app, “FoodSwitch”, allowed consumers to identify foods packaged with less salt and was available for free download [39].

Takada et al. [19] described an intervention with cooking classes. The 90-min classes were held twice and consisted of a practical course for evaluating the amount of salt in a meal and instruction on salt-reduced cooking.

Markota et al. [32] provided warning stickers about the harmful effects of excessive salt to be affixed to all salt containers, as well as providing individual information leaflets received on the undesirable effects of excessive salt consumption [32].

Yokokawa et al. [29] described an education program with visualization tools to inform participants about their estimated salt intake and health education classes. Participants provided a sample of their soup three times over the course of the study, and researchers reported the amount of salt the soup contained, measured using a digital handheld device. In addition, participants were informed of their sodium excretion. The education classes involved participants in reducing their daily dietary salt intake, suggesting ways to prepare tasty, low-salt meals [29].

In the study by Anderson et al. [34] before starting the intervention, all participants consumed a controlled low-sodium diet for 4 weeks. Participants received all foods, snacks and beverages that contain calories. After the controlled diet, participants in the intervention group were asked to continue eating a low-sodium diet. Participants had one-on-one counseling sessions, by phone or email, and group counseling sessions that included cooking demonstrations and received spices and a cookbook. Participants monitored their sodium intake through a sodium-specific tracking tool that allowed them to record the foods, brand name and description, and the amount of sodium consumed [34].

#### 3.3.4. Effects of Interventions

In the study by Cashman [30] et al. the intervention showed a decrease in salt intake of 1.7 g/day, *p* < 0.0001 on average during the low-salt diet period. Systolic blood pressure was significantly lower (3.3 mmHg on average, *p* < 0.0001) and there was no statistically significant difference in diastolic blood pressure.

Morikawa et al. [22] showed that the mean daily salt excretion decreased 0.7 g/day (from 11.5 ± 1.8, *p* = 0.008) in intervention group. Mean diastolic blood pressure decreased by 6.2 mmHg (*p* < 0.001) in the intervention group and between groups the difference was 4.5 mmHg (*p* = 0.012). Systolic blood pressure decreased by 5.4 mm Hg in the intervention group (*p* = 0.012), with no significant difference between groups.

Land et al. [39]. reported that estimated mean salt intake decreased by around 0.8 g/day (from 8.8 ± 3.6 g/day, *p <* 0.001.

In the study by Riis et al. [31] mean salt intake decreased by 1.8 g/day (from 9.25 ± 2.5, *p* < 0.001) in adults in intervention group A and in intervention B it decreased by 1 g/day (from 9.5 ± 3.0, *p* = 0.085). The mean difference between groups in intervention A was −0.5 g/day (*p* = 0.523) in intervention B it was −1 g/day/day (*p* = 0.079).

In the study by Barros et al. [37] mean salt intake decreased by around 4.5 g/day (from 11.8 ± 7.6 mg/day in the intervention group and the mean difference between groups was −3.2 g/day (*p* = 0.023). Systolic blood pressure and diastolic blood pressure differed significantly between the intervention group and the control group, 12.47 mmHg, *p =* 0.034 and 7.58 mmHg, *p =* 0.046, respectively.

In the study by Li et al. [28] the mean difference in salt intake between groups was 0.8 g/day. In villages with price subsidy the mean salt intake was 13.34 ± 5.5 g/day and without price subsidy was 14.0 ± 5.6 g/day. There was no significant difference in blood pressure.

Jafari et al. [26] described that mean salt intake decreased by around 0.9 (from 8.8 ± 0.2 g/day, *p* = 0.001) in the intervention group. Systolic blood pressure decreased by around 7.4 mmHg in the intervention group. There were no significant differences in diastolic blood pressure.

The intervention described by Yokokawa et al. [29] described that mean salt intake decreased by around 0.86 g/day (from 10.0 ± 2.2 g/day, *p* < 0.01) in the intervention group and the adjusted difference between the intervention group and the control group was −0.66 g/day (*p* = 0.03) at 6 months. At 12 months, salt intake decreased by 0.22 g/day (*p* = 0.02) and the mean difference between groups was −0.42 g/day, but it was not significant (*p* = 0.16). Systolic blood pressure decreased by around −7.55 mmHg (*p* < 0.01) between groups after adjusting for covariates at 6 months. These differences were not observed at 12 months. There were no differences in diastolic blood pressure.

Takada et al. [19] reported that mean daily salt intake decreased by 0.57 g/day (from 9.57 ± 2.45 g/day) in the intervention group and the mean difference between groups was −1.16 g/day (*p* = 0.033). There was no effect on blood pressure.

In the study by Anderson et al. [34] during controlled consumption (phase 1), salt intake decreased by 4.6 g/day. At the end of the intervention (phase 2), the mean difference between groups was −2.4 g/d (*p* = 0.002) after controlling for sodium intake at screening.

Markota et al. [32] reported that the mean salt intake decreased by around 2.0 g/day (from 12.1 ± 4.9 g/day, *p* < 0.0001) in the intervention group. Systolic blood pressure decreased around by −5.3 mmHg (*p* < 0.0001) and diastolic blood pressure decreased around by −2.9 mmHg (*p* < 0.0001) in the intervention group.

#### 3.3.5. Estimates of Salt Intake

The intervention to reduce salt intake in three interventions was to provide tools for participants to estimate salt intake: an app (*n =* 1) [36] and a self-monitoring device for urinary sodium excretion (*n =* 2) [20,23].

Ipjian et al. [36] used a “MyFitnessPal” application to reduce participants’ salt intake. The profile of participants in the app was programmed by the investigator to a sodium level of 2300 mg/d. Verbal and written instructions were given about using the app, and participants were instructed to use the app daily for food and beverage input to monitor dietary sodium levels [36].

Both authors, Takada et al. [20] and Yasutake et al. [23] described asking participants to measure daily salt excretion at home for 4 weeks using the self-monitoring device.

#### 3.3.6. Effects of Interventions

In the study by [36] Ipjian et al. [36] mean salt intake decreased by around 2.1 g/day (from 10.46 ± 5.27 g/day) in the app group.

In the study by Takada et al. [20] mean salt intake decreased by around 0.77 g/day (from 9.37 ± 2.13 g/day) in the intervention group. The mean difference between the two groups was −0.50 g/day (*p =* 0.030). The mean difference between the two groups was −4.4 mm Hg in systolic blood pressure.

In the study by Yasutake et al. [23] salt excretion decreased at weeks 3 and 4 by around 0.07 g/day (from 8.31, *p* < 0.059 and 0.2 g/day (from 8.24, *p<* 0.01), respectively. In total, from baseline to the end of the study decreased by 0.2 g/day. Diastolic blood pressure decreased by around 3.4 mmHg (*p <* 0.05). There were no differences in diastolic blood pressure.

### 3.4. Risk Ob Bias in Studies

**Figure 2:**
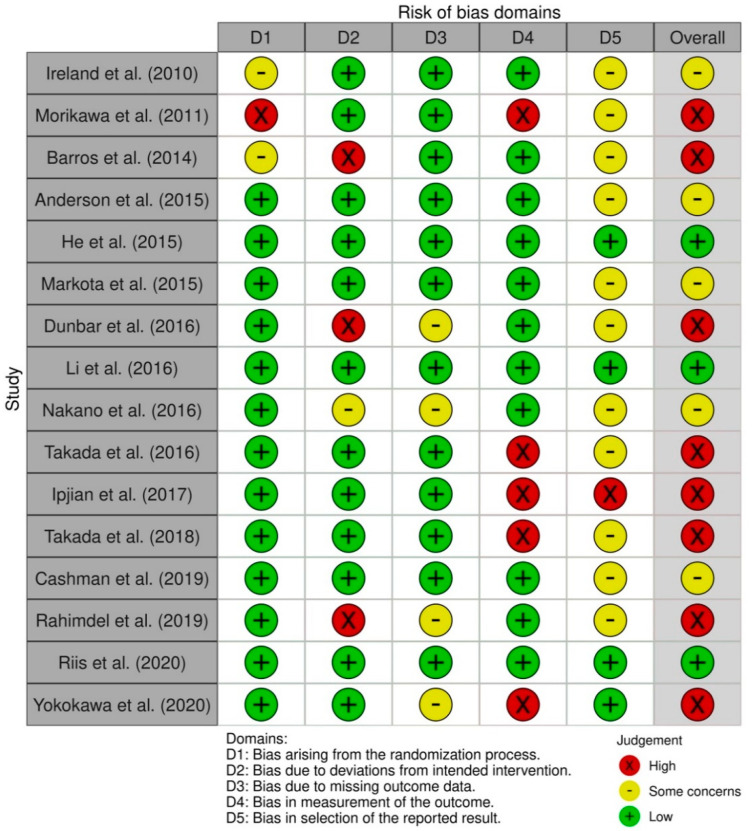
Risk of bias summary for randomized controlled trials (Cochrane risk of bias tool (RoB 2)).

In randomized controlled trials, three studies had a low overall risk of bias [27,28,31], five studies showed some concerns about the overall risk of bias [21,30,32,34,38] and eight studies had a high overall risk of bias [19,20,22,24,29,35,36,37]. Most studies had a low risk of bias in bias arising from the randomization process, with the exception of three [22,37,38]. The bias in selection of the reported result was the one with the most concerns about the risk of bias (*n =* 11) [19,20,21,22,24,30,32,34,35,37,38] and one had a high risk of bias [36] (Figure 2).

**Figure 3:**
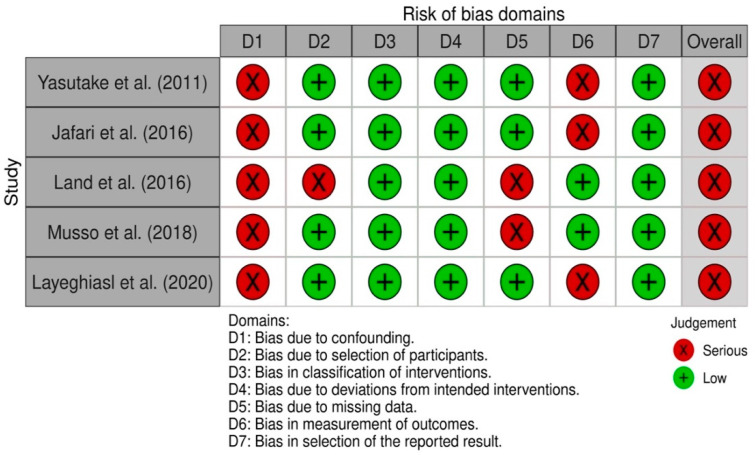
Risk of bias summary for nonrandomized studies (ROBINS-I tool).

All nonrandomized intervention studies had a serious risk of global bias [23,25,26,33,39]. The five non-randomized studies had low risk of bias in classification of interventions, bias due to deviations from the intended interventions, and bias in selection of the reported outcome. All studies had severe risk bias due to confounding (Figure 3).

## 4. Discussion

This review identified 21 recent interventions that successfully reduced salt intake. It provides evidence that interventions based on individual education, with or without other associated interventions and tools to estimate salt intake, have positive results in reducing salt intake. However, the analyzed studies must be interpreted with care due to the mixed quality of the study designs, the different interventions and the lack of some intervention details, in addition to the difficulty in identifying specific characteristics of the interventions that led to success in reducing salt consumption.

Health and nutrition education interventions appeared to be the ones that achieved the greatest salt reduction, with salt reduction ranging from about 0.9 g/day to 4.7 g/day. Salt reduction in nutritional education, plus other interventions, ranged from about 0.57 g/day to 4.5 g/day. Estimates of salt intake interventions reduced salt intake between about 0.4 and 2.1 g/day. However, it is important to keep in mind that the mean baseline sodium value varied widely across all interventions. Reductions in higher basal values seems to can reach greater magnitudes; however, these data must be interpreted with caution.

The understanding of the underlying mechanisms and causes of chronic diseases is transforming medicine from a reactive discipline to a proactive and preventive one. Therefore, a Predictive, Preventive, Personalized and Participatory Medicine (medicine P4) [40] which also applies to nutrition, with the objectives of quantifying well-being, will predict and prevent disease. Consumers are different in each country and the form of salt consumption is also different.

Tailoring treatment to each person’s characteristics means classifying others into subpopulations that differ in their susceptibility to a specific intervention or in their response to a specific treatment. Preventive interventions can then be focused on those who will benefit, saving resources for those who will not [41]. This approach reflects the importance of interventions that are based on the individual and not the general population.

We identified three randomized studies with low-risk of bias that successfully reduced adults salt intake: dietary education and availability of a salt substitute with added potassium at village shops [28]; salt reduction in bread and nutritional advice [31]; and through the nutritional education of children who delivered the message to their families [27]. A reduction in salt intake is possible by integrating salt reduction education modules into school curricula and empowering children to deliver the message of salt reduction to their families. This intervention showed a new, viable and effective approach to reducing salt intake, and the authors showed an approximately 25% reduction in salt intake by the participants and a decrease in systolic blood pressure. Passing a salt reduction message to children has the potential to establish habits and attitudes that will persist throughout adult life, in addition to being able to reduce the consumption of salt by the family as well. It is an intervention that can be customized according to the children of each school. The authors report that to achieve a greater reduction in the population’s salt intake, this approach must be combined with other strategies such as working with the food industry to gradually reduce the amount of salt added to all processed foods [27].

The family also plays an important role in reducing salt intake in patients with heart failure [35], as family members can provide motivation and positive communication to change family habits [42].

Providing low-salt bread is an effective salt reduction strategy combined with nutritional counseling [26,30,31]. By reducing salt added to bread or other foods, the specific salt taste receptors in the mouth become much more sensitive to lower salt concentrations, meaning that less salty foods will stimulate a sensorial response similar to very salty foods before the adjustment [43]. This intervention can be one of the solutions for reducing salt in places where processed foods are one of the main sources of salt intake.

The use of salt substitutes with lower sodium content was a strategy used in interventions by three studies in this review [28,37,39]. Salt substitute is effective in reducing salt intake and has potassium in its constitution, increasing potassium intake by consumers. In the studies included in this review, potassium intake was higher in the intervention groups, but participants did not reach the WHO recommended daily intake (3510 mg/day). Also, a recent study showed that the use of salt substitute (with potassium in the constitution) decreased urinary sodium excretion and the rates of stroke, cardiovascular events, and deaths from any cause in people over 60 years of age [44].

The use of substitute salt costs about twice as much as regular salt [28], and it can be a social barrier in countries with less economic power. In addition, it has been reported that the taste of the salt substitute is bitter, not being accepted by all consumers [37]. It is important to consider whether dietary education focused on reducing salt intake can be a better and cheaper strategy that is more integrative from a social point of view. This review included seven interventions focused only on educating the individual to reduce salt intake and customized according to the target audience or their consumption habits. These interventions were successful and do not depend on the economic power of the consumer to buy a substitute that will make them ingest less salt.

In the study by Land et al. [39] the salt substitutes were part of a multifaceted community-based salt reduction program. As the intervention consisted of several components that were implemented simultaneously, it is not possible to quantify the relative contributions of each one to the success of the program. The same was found in the interventions by Anderson et al. [34] and Layeghiasl et al. [25], and it is not possible to identify a factor as being more important than another in the success of the intervention. Changing eating behavior is a complex process and dealing with multiple and interrelated factors appears to be effective in reducing salt intake [34]. The World Health Organization defends that different approaches can be applied in health education and communication campaigns; namely social mobilization, social marketing, behavior change communication and communication for development [12].

This review includes interventions where self-monitoring appears to be effective in behavior change and an effective complementary strategy in salt reduction [20,22,23,36]. There is a growing trend towards self-monitoring of health, especially through apps such as calorie counters, exercise or dietary advice, and there is evidence that self-monitoring benefits users. Monitoring has the advantage that users can share dietary monitoring with healthcare professionals and receive immediate feedback or long-term follow-up [45]. It is a patient-centered and personalized service, making it possible to verify the individual’s salt intake and to be able to define strategies to reduce salt intake [36,46].

Maintaining the effects of salt-reducing interventions is difficult over time [47]. Sustainable changes in consumer behavior seem to be achievable through knowledge and awareness. Much of the population is not aware of the risks of salt consumption and its relationship with hypertension and its comorbidities. In addition to not being aware of the maximum recommended daily dose of salt intake, the amount of salt they eat and the main sources of salt in their diet [12].

The intervention based on the theory of planned behavior included in this review reduced salt intake by about 35% in the intervention group [24]. This approach is interesting in reducing salt consumption, as it customizes the intervention for each individual and there are different attitudes, stimulants and inhibitors about salt consumption in the population, in addition to several variables that affect the person’s control over behavior. However, a central factor in the theory of planned behavior is the individual’s intention to perform a certain behavior [48]. In the study by Rahimdel et al. [24] participants were at risk of developing hypertension, which can lead to being more motivated to change behavior than healthy individuals, which shows us that it is important to customize interventions according to the group target.

We have also seen success in reducing salt intake in interventions based on nutrition education with a nutritionist [21,33], educating the consumer to read labels to select healthier foods that contain less salt [32] and cooking classes given by health professionals, including a nutritionist [19]. Having a health professional who can teach and raise awareness about the impact of salt consumption on health and the main sources of salt in the diet seems to help influence consumer behavior [12].

This review only included interventions that reduced salt intake with statistical significance (*p* < 0.05). When searching for interventions that were successful in reducing salt intake, we found 11 interventions that either had no statistically significant salt reduction or did not reduce salt intake. Summary tables on these interventions can be found in Supplementary Materials (Supplementary Tables S1 and S2). These interventions included self-monitoring of salt excretion (*n* = 2) [49,50] and Na:K ratio (*n* = 1) [51] in urine. Self-monitoring of Na:K ratio excretion reduced salt excretion without statistical significance, probably because the sample size was insufficient and baseline potassium excretion was greater than the authors had expected. Also, in interventions with self-monitoring of salt excretion there was a non-statistically significant decrease in salt, the authors reported that this was probably due to a short intervention period (4 weeks) and insufficient sample size. Although these interventions were not included as successful interventions, it is likely that if they did not have problems with the methodology, they could have been successful. Participants being able to estimate salt intake appear to be effective salt reduction strategies as mentioned in other interventions [20,23] included in this review. A nutrition education intervention was unsuccessful in reducing salt, the intervention was to teach diabetic participants to use the nutrition information panel on food labels to choose products that comply with the Food Standards Australia New Zealand (FSANZ) guideline of <120 mg sodium/100 g food [52]. This intervention was used by Ireland et al. [38] in free-living adults and have successfully reduced salt. Therefore, this type of intervention is not effective in diabetics, which reinforces the importance of customizing interventions according to the population. We found three interventions that, in addition to nutrition education, used apps to reduce salt intake. Two interventions reduced salt without statistical significance and one intervention failed to reduce salt. Dorsch et al. [53] described an application-based intervention that sends just-in-time contextual adaptive messages. The reduction in urinary sodium excretion was 637 mg/day, but without statistical significance. Although the authors report that there were clinically significant improvements in the intervention group compared to the control, all participants were required to have an iPhone, so the effectiveness of this intervention may be related to the socioeconomic status of the participants. Lofthouse et al. [54] described an intervention that, in addition to using the app, used salt substitutes with lower sodium content, participants reduced salt excretion by 433 mg/day without statistical significance. This was a pilot study with only 11 volunteers, and these had a low baseline sodium (2342 mg) and so we probably could not see the potential of this intervention to reduce salt intake. In the study by Thatthong et al. [55] they described an intervention using a program that sends interactive messages about salt reduction. The study was carried out in hypertensive patients, at the end of the study, sodium excretion in the intervention group was higher than the baseline value. Although the sample size was small (*n* = 50), this result indicates that this intervention is probably not effective in reducing salt intake in hypertensive patients. Nakadate et al. [56] described an intervention in which they provided a salt monitoring instrument to measure the salt concentration of soup at home and low-sodium seasoning. They achieved a sodium reduction of 777 mg/day with monitoring and 413 mg/day with the low-sodium seasoning. Although the results were not statistically significant, probably due to the exploratory pilot design of the study, with sample size calculated based on provisional statistics, the results are interesting, especially the monitoring of salt in soup, in regions that have a high consumption of soup. Another study described an environmental and behavioral intervention in the workplace. They achieved an average reduction in salt intake of −0.6 g, from 8.7 g but without statistical significance. The authors reported that the cause of not achieving greater salt reduction was poor adherence to the study and programs in catering operations. The authors concluded that acceptance, effectiveness, and maintenance of workplace nutrition interventions require strong employer support [57]. Therefore, it is important to only consider intervening in the workplace when the employer is motivated to reduce the salt intake of workers. We found two studies that used Salt-Restriction-Spoon in the intervention. Chen et al. [58] in addition to the spoon, they provided nutritional education and informed the participants of the value of sodium excretion. At the end of the intervention, both the control group and the intervention group had decreased sodium excretion without statistical significance. Participants in both groups lived in the same place, probably causing contamination of the information for the study, the participants in the control group were informed about their sodium excretion, which may have contributed to the reduction in sodium excretion in this group. Cornélio et al. [59] described an intervention in hypertensive women that, in addition to the use of the Salt-Restriction-Spoon, provided an education based on behavior modification techniques to reduce salt intake. Also, in this intervention, both the control group and the intervention group decreased sodium excretion without statistical significance. Although the authors did not mention it is possible that there was an influence to reduce salt consumption in the control group because the women were asked to assess their usual monthly salt intake, and this may have led to awareness of the amount of salt they used and led them to reduce the amount they used when cooking. Salt-Restriction-Spoon are very interesting in populations where the biggest source of salt is the addition to cooking, helping people to limit the addition of salt.

In these 11 interventions that were not included in the review, we were able to perceive that they had no effect either for methodological reasons or because they had no effect on a particular population, reinforcing the importance of adapting interventions to reduce salt.

The inclusion of studies that only analyzed salt intake by urinary excretion could be pointed to as a strength of this review. The gold standard method is 24-h urinary excretion, as approximately 90 to 95% of ingested sodium is excreted in the urine [60]. However, we have included all studies that estimated salt intake through urinary measures such as spot urine collection and overnight urine sample, as they were previously described as measures to estimate acceptable salt intake [61,62,63,64,65].

## 5. Conclusions

Consumer education-based interventions alone reduce salt intake, but also when combined with other strategies. Tools for estimating salt consumption and self-monitoring of its consumption are also successful in reducing it.

In this review there is no evidence that the type of intervention analyzed is more effective in reducing salt consumption, but according to the medicine P4 approach, we must analyze each revised intervention and verify in which individuals or subpopulations it is most beneficial and will lead to better results. However, the results must be interpreted with caution as the quality of the studies is mixed. In the future, it is important to develop more high-quality clinical trials, with a longer intervention time and more participants, in order to understand which interventions work best for the reduction of salt consumption according to the target population.

## Figures and Tables

**Table 1:** Overview of characteristics of the included randomized trials.

Study Author (Year), Country	Participants	Study Design	Enrolment Start and End Dates; Length of Participant Follow-Up	Intervention	Method of Assessment	Results		
						Reduction in Salt Intake (g/day) in the IG	Difference in Salt Intake (g/day) between IG and CG	Blood Pressure (mmHg)
Ireland [38] (2010), Australia	43 Healthy free-living adults (33 women). Tick group, *n =* 22. Food Standards Australia New Zealand group, *n =* 21.	8-week parallel design randomized	August 2008 and October 2008.	Participants received dietary education to choose foods identified by either Australia’s National Heart Foundation Tick symbol or by the Food Standards Australia and New Zealand’s low-salt guideline of 120 mg sodium/100 g food.	24 h urinary Na excretion, 1 urine collection at 3 different times (baseline, week 4, week 8).	0.9 in Tick group 2.0 in Food Standards group	NI	NA
Morikawa [22] (2011), Japan	41 Hypertensive male workers employees of a rail-road company. IG, *n =* 22, (48 mean years). CG (*n =* 19, 47 mean years).	Quasi-randomization intervention control	4-week follow-up.	Self-monitoring of daily salt excretion by an electronic salt sensor and sent personalized e-mail advice via cellular phone.	Overnight urine using the electronic salt sensor, only participants in IG (for 1 week at baseline and during the week 4). BP (baseline and week 4)	0.7	NA	SBP: −5.4 in IG DBP: −6.2 in IG
Barros [37] (2014), Brazil	38 Hypertensive individuals (56 mean years, 65.7% women). IG, *n =* 19. CG, *n =* 16.	Single-blind randomized controlled trial	May to October 2012, 4-week intervention.	All patients were instructed to consume only the provided salt throughout this study (light salt for the intervention group and regular salt for the control group). In addition, they were instructed to reduce sodium-rich food consumption during the study period, being particularly warned about industrialized foods.	24 h urinary Na excretion, 1 urine collection at 2 different times (baseline and at the end of the trial). BP (baseline and at the end of the trial).	4.5	−3.2	SBP −12.47 (*p* = 0.034) in IG×CG. DBP −7.58 (*p* = 0.046) in IG×CG
Anderson [34] (2015), USA	40 Individuals for whom the Dietary Guidelines for Americans recommends 1500 mg Na/day (61 mean years, 88% African American). IG, *n =* 20. CG, *n =* 20.	Randomized clinical trial	2012 to 2014, 20-week intervention.	2-phase study. In phase 1, all participants consumed a low-sodium diet for 4 weeks. Participants were provided all foods, snacks, and calorie-containing drinks. In phase 2, were randomly assigned to either a multifactorial behavioral intervention emphasizing spices and herbs to reduce sodium intake or a self-directed control group.	24 h urinary Na excretion, 7 urine samples as follows: 2 samples at screening, 2 samples at week 4 (baseline), one sample at week 14, and 2 samples at week 24.	2.3	−2.4	NA
He [27] (2015), China	553 Adult family members of primary school children. CG, *n =* 275 (44 mean years). IG, *n =* 278; 44 mean years.	Cluster-randomized controlled trial	May to December 2013, 3.5-month intervention.	Children in the intervention group were educated on the harmful effects of salt and how to reduce salt intake within the schools’ usual health education lessons. Children then delivered the salt reduction message to their families. Parents were provided with educational materials in the form of a newsletter.	24 h urinary Na excretion, 2 urine collections at 2 different times (at baseline and at the end of the trial). BP (at baseline and at the end of the trial).	2.1	−2.9	SBP −2.3 (*p* < 0.05) in IG×CG. DBP −0.9 (*p* = 0.31) in IG×CG
Markota [32] (2015), Bosnia-Herzegovina	150 Adults treated hypertensives. CG, *n =* 74 (59 mean years, 37 women). IG, *n =* 76 (59 mean years, 40 women).	Randomized clinical trial	September 2012 to July 2013	Intervention group: received individual information leaflets about the untoward effects of excessive salt consumption and received warning stickers to be mounted on all salt containers. Control group: only information leaflets.	24 h urinary Na excretion, 1 urine collection at 3 different times (baseline; 1 month and 2 months after the intervention). BP (baseline, 1 month and 2 months after the intervention).	2.0	NI	SBP: −5.3 in IG DBP: −2.9 in IG
Dunbar [35] (2016), USA	117 Heart Failure patients and one family member–dyads (56 mean years, 37% women). Usual Care (UC), *n =* 37. Family Education (PFE), *n =* 41. Family Partnership Intervention (FPI), *n =* 37.	3-Group randomized control trial	4-Month behavior change. 4 to 8-month maintenance phase of behavior change.	UC group: received usual care from their providers and was provided with educational pamphlets that were created by the Heart Failure Society of America (HFSA). PFE group: received usual care, the HFSA pamphlets, and educational sessions. FPI group: received the same education and counseling as described in the UC and PFE groups plus 2-additional group sessions that focused on teaching the dyads how to give support, communication, empathy, and autonomy support for one another’s roles.	24 h urinary Na excretion, 1 urine collection at 3 different times (baseline, 4 and 8 months after the intervention).	3.1 in FPI	NI	NA
Li [28] (2016), China	120 Townships from five provinces (*n =* 1903). Control villages, *n =* 60. Intervention villages, *n =* 60.	Two parallel cluster-randomized trial.	May 2011 to November 2012. 18-month intervention.	Intervention villages-Community-based health education and availability of reduced-sodium, added-potassium salt substitute at village shops. Control villages-Continued their usual practices	24 h urinary Na excretion, 1 urine collection in a single moment (end of intervention). BP (end of intervention).	NI	−0.8	SBP: −1.1 (*p* = 0.33) in IG×CG. DBP: −0.7 (*p* = 0.35) in IG×CG
Nakano [21] (2016), Japan	95 Hypertensives IG, *n =* 51 (35 women, 58 mean years). CG, *n =* 44 (24 women, 60 mean years).	Prospective, randomized, and open-label study	September 2012 to May 2014, 3-month follow-up.	Intervention: intensive nutritional education aimed at salt restriction to 6 g/d by nutritionists. Control: conventional salt-restriction education.	24 h urinary Na excretion, 1 urine collection at 2 different times (baseline and after intervention). Monitoring of clinic, home, and ambulatory BP values (baseline and after intervention).	1.8	NI	SBP: −4,5 in IG (Ambulatory 24 h SBP)
Takada [19] (2016), Japan	35 Housewife’s and 31 family members. IG, *n =* 36 (63 mean years, 20 women). CG, *n =* 32 (65 mean years, 22 women).	Single-blinded, family-based Cluster randomized controlled trial	September 2015 to October 2015, 2-month intervention.	Intervention Group: 2x Cooking classes by registered dietitians, a general physician and a nephrologist, and it consisted of a practical course for evaluating the amount of salt in a meal and instruction on salt-reduced cooking. Control group: participants attended lectures about healthy living. The lecture contents did not include information related to salt reduction.	Spot urine, 1 urine collection at 2 different times (baseline and 2 months after intervention) BP measured only in the housewife’s subgroup (baseline and 2 months after intervention	0.57	−1.16	SBP: −3.6 (*p* = 0.371) in IG×CG. DBP: −1.68 (*p* = 0.606) in IG×CG
Ipjian [36] (2017), USA	30 Healthy adults (34 mean years; *n =* 23 women). MyFitnessPal app group, *n =* 15. Journal group, *n =* 15.	Randomized parallel trial	August to December 2014, 4-week intervention.	Participants were instructed to reduce their sodium intake to ≤2300 mg/d by using the MyFitnessPal app to receive feedback on sodium content of foods or by paper tallying of estimated sodium intake.	2 first morning spot urine collection at 2 different times (baseline and after intervention). BP (baseline and after intervention)	2.1	NI	NI
Takada [20] (2018), Japan	158 Participants from 105 families. IG, *n =* 79 (61 mean years, 68.4% women). CG, *n =* 79 (64 mean years, 64.6% women).	Single blinded, family-based, cluster randomized controlled trial	September 2016 to April 2017. 4-week intervention	Participants in both the intervention and control groups attended lectures about salt reduction by a general physician and a registered dietitian. In the intervention group, participants used the self- monitoring device to estimate their daily salt intake, and they recorded their results for 4 weeks.	Spot urine, 1 urine collection at 2 different times (baseline, week 4) BP (baseline, week 4).	0.77	−0.50	SBP: −4.4 in IG×CG
Cashman [30] (2019), Republic of Ireland	46 Adults with slightly to moderately elevated BP (47 mean years, 40 women)	Randomized crossover trial	January 2008 to July 2010. 5-week intervention.	Intervention: Combination of pragmatic dietary advice with the replacement of bread and a limited number of other foods with equivalent foods with a lower salt content. Control: normal diet, but were asked to consume an in-house bread, equivalent in composition to the low-salt version, but with its more typical salt content.	24 h urinary Na excretion, 1 urine collection at 3 different times (baseline, week 5, week 10) BP (baseline, week 5, week 10)	1.7	NI	SBP: −3.3 in IG
Rahimdel [24] (2019), Iran	140 Adults at risk of developing hypertension (43 mean years, 59.3% women). IG, *n =* 70. CG, *n =* 70.	Randomized clinical trial	February 2017 to December 2017	Intervention: Education program based on the theory of planned behavior (TPB) for salt intake in individuals at risk of hypertension. Based on the results of the pretest, the educational content was prepared in the form of a booklet. Control: No educational program was conducted for the control group.	24 h urinary Na excretion, 1 urine collection at 2 different times (baseline and 2 months after the intervention) BP (baseline and 2 months after the intervention).	4.7	NI	SBP: +1.1 (*p* = 0.2) in IG DBP: +1.26 (*p* = 0.22) in IG
Riis [31] (2020), Denmark	Family with at least one child. IG A, *n =* 41 (42 mean years). IG B, *n =* 63 (41 mean years). CG, *n =* 49 (41 mean years).	single-blinded, cluster randomized controlled trial with a parallel design	January 2018 to July 2018, 4-month intervention.	Intervention A: Families received sodium-reduced bread Intervention B: Families received sodium-reduced bread and dietary counseling Control: Families received regular sodium bread	24 h urinary Na excretion, 3 urine collections at 2 different times (baseline and 4 months after the intervention).	1 in IG A (*p* = 0.085) 1.8 in IG B (*p* < 0.001)	−0.5 (IG A x CG), *p* = 0.523 −1.0 (IG B x CG), *p* = 0.079	NA
Yokokawa [29] (2020), Thailand	753 Patients at high risk of CVD stratified by the Framingham general CVD risk scoring system (367 women). 8 clusters: *n =* 4 CG, *n =* 4 IG.	cluster randomized controlled trial	February 2012 to January 2013	Intervention group: education program, visualization tools to inform the patients of their estimated 24-h salt intake and dietary salt content in daily food/ soup (analyzed by investigators) and special health education classes organized by the dietician. Control group: routine care services and a brief individual health education session, not focused on salt reduction.	Overnight urine collection, average of 3 successive days’ measurements at 3 different times (baseline, 6 and 12 months). BP (baseline, 6 and 12 months)	0.86 at 6 months (*p* < 0.01) 0.22 at 12 months (*p* = 0.02)	−0.66 at 6 months (*p* = 0.03) −0.42 at 12 months (*p* = 0.16)	SBP At 6 months in IG: −12.34 IG×CG: −7.55 SBP

NI—No information; NA—Not applicable; BP—Blood pressure; SBP—Systolic blood pressure; DBP—Diastolic blood pressure; IG—Intervention Group; CG—Control group.

**Table 2:** Overview of characteristics of the included nonrandomized trials.

Study Author (Year), Country	Participants	Study Design	Enrolment Start and End Dates; Length of Participant Follow-Up	Intervention	Method of Assessment	Results		
						Reduction in Salt Intake (g/day) in the IG	Difference in Salt Intake (g/day) between IG and CG	Blood Pressure (mmHg)
Yasutake [23] (2011), Japan	30 Healthy adult volunteers (15 women, 43 mean years).	Quasi-experimental	March to April 2009, 4-week intervention.	Measurement of daily salt excretion at home for 4 weeks using the self-monitoring device for educating healthy adults regarding their levels of salt intake and the dangers of excessive salt use.	Overnight urine sample, 4 weeks using the self-monitoring device. BP (baseline and 8 weeks later).	0.4	NA	SBP: −3.4
Jafari [26] (2016), Iran	Two cities. Intervention, *n =* 346 (61% women, 49 mean years). Control, *n =* 310 (50.3% women, 48 mean years).	Community intervention trial	March to July 2014, 4-week intervention.	Installation of educational banners and door-to-door distribution of pamphlets in the intervention city and in the control city. In the intervention city, they reduced the bread salt by 40%	Urine sample collection from 8:00 to 9:00 am after discarding the first urine, 1 urine collection at 2 different times (baseline and after 12 weeks). BP (baseline and after 12 weeks).	0.9	NA	SBP: −7.4 in IG
Land [39] (2016), Australia	419 individuals at baseline and 572 at follow-up (56 mean years, 58% women).	Interventional (Clinical Trial)/Community-based intervention	March 2011 to May 2014, 18-month intervention.	A multi-faceted, community-based salt reduction program using the Communication for Behavioral Impact (COMBI) framework.	24 h urinary Na excretion, 1 urine collection at 2 different times (baseline and at the end of the intervention)	0.8	NI	NA
Musso [33] (2018), Italy	291 Patients on antihypertensive treatment (166 women, 63 mean years). Dietary protocol *n =* 240, control *n =* 51.	Intervention control Trial	2-month intervention.	Low-sodium diet prescribed by the dietitian.	24 h urinary Na excretion, 1 urine collection at 2 different times (baseline and after intervention). BP (baseline and after intervention).	1.14	NI	SBP: −7.66 in IG DBP: −4.69 in IG
Layeghiasl [25] (2020), Iran	166 participants (50% women, 36 mean years).	Quasi-experimental pretest-posttest with control group design	2-month intervention.	Intervention: Marketing mix components were determined for designing an intervention. An educational package focused on reducing salt intake and using alternatives was developed. Control: received routine interventions in healthcare centers.	Urine samples collected daily in the morning, 1 urine collection at 2 different times (baseline and at the end of the intervention).	3.01	NI	NA

NI—No information; NA—Not applicable; BP—Blood pressure; SBP—Systolic blood pressure; DBP—Diastolic blood pressure; IG—Intervention Group; CG—Control group.

## Data Availability

Not applicable.

## Supplementary Materials

The following are available online at https://www.mdpi.com/article/10.3390/nu14010006/s1, Table S1: General characteristics of randomized trials without statistically significant salt reduction (*p* < 0.05), Table S2: General characteristics of non-randomized studies without statistically significant salt reduction (*p* < 0.05).
