# Supplementary material for: Interventions That Successfully Reduced Adults Salt Intake—A Systematic Review
Source: Nutrients. 2021 Dec 21;14(1):6. doi: 10.3390/nu14010006 (PMC8746410; doi:10.3390/nu14010006)
Supplement: Supplementary file 1 [file nutrients-14-00006-s001.zip › nutrients-1476056-supplementary.pdf]

Table S1. General characteristics of randomized trials without statistically significant salt reduction (p<0.05)

Study author (year), country	Participants	Study design	Enrolment start and end dates; length of participant follow-up	Intervention	Method of assessment	Results
Petersen (2013), Australia	78 patients with type 2 diabetes. Intervention group, *n*=39 (13 women, 63 mean years). Control group, *n*=39 (16 women, 62 mean years).	Randomized control trial	3-month intervention.	Nutritional education in a single session to use the nutrition information panel on food labels to choose products which complied with the Food Standards Australia New Zealand (FSANZ) guideline of <120 mg sodium/100 g food.	24h urinary Na excretion, 1 urine collection at 2 different times (baseline and after intervention). BP (baseline and at the end of the intervention).	After intervention mean urinary sodium excretion was unchanged in the intervention (174 ± 13 mmol/day vs 175 ± 13 mmol/day) and there was no between group difference (p > 0.05). No effect on blood pressure.
Chen (2013), China	403 adults responsible for home cooking. Intervention group, *n*=226 (75.2% women, 55 mean years). Control group, *n*=177 (67.8% women, 52 mean years).	Community intervention study.	6-month intervention. June 2012 to January 2013.	Salt-restriction-spoon and health education, and participants were informed of actual salt intake and 24-hour urinary Na excretion.	24h urinary Na excretion, 1 urine collection at 4 different times (baseline, visit 2, visit 3 and at the end of the intervention).	The urinary Na excretion decreased by 34.84 mmol in the intervention group and by 33.65 mmol in the control group, and repeated measures analysis of variance showed no difference between groups by time (F=0.222, P=0.870).
Cornélio (2016), Brazil	92 Hypertensive women. Intervention group, *n*=43 (59 mean years). Control group, *n*=49 (61 mean years).	Randomized controlled pilot study	2-month intervention.	The intervention was based on behavior change techniques to motivate participants to reduce salt addition, increasing self-efficacy and neutralizing the negative influence of the habit. It included guided practice, with practical skills-building activities, using a measuring spoon for 4 g of salt; and counter-conditioning activities, employing the presentation of natural spices as an alternative to the use of salt.	24h urinary Na excretion, 1 urine collection at 2 different times (baseline and at the end of the intervention).	Mean 24-hour urinary salt difference between groups was 0.35 (p < 0.56; d = 0.51). At baseline, mean salt intake was 10.7 g/day in both groups. After the intervention, salt consumption decreased in both groups, in the intervention group it decreased to 9.1 g/day and in the control group to 9.6 g/day. There were no significant differences between groups.
Nakadate (2018), Japan	50 healthy adults. Intervention group, *n*=28 (67.9% women, 56 mean years). Control group,	Double-blind randomized controlled study using a	November 2013 to August 2014.	The study used a 2 × 2 factorial design with two types of intervention: (1) monitoring the concentration of salt in homemade dishes and (2) using a low-sodium seasoning regardless of the amount. The monitoring	24h urinary Na excretion, 1 urine collection at 2 different times (baseline and at the end of the intervention).	Sodium intake between the intervention and control groups did not statistically differ (−1011 mg/day and −283 mg/day for Monitoring and Seasoning, respectively).

	n=22 (72.7% women, 50 mean years).	2 × 2 factorial design with two interventions		group received a measuring instrument (digital salt meter model SK-5SII; Sato Keiryoki Mfg. Co., Ltd., Tokyo, Japan) to measure the salt concentration of home-cooked soup dishes.		
Iwahori (2018), Japan	92 Free-Living adults	Randomized controlled trial	1-month intervention. January 2012 to March 2012.	The intervention was self-monitoring of the urinary sodium-potassium ratio using monitors (HEU-001F, OMRON Healthcare Co., Ltd., Kyoto, Japan). The intervention group was asked to measure at least a daily urine Na:K ratio and advised to reduce their Na:K ratio towards the goal of less than 1.	24h urinary Na excretion, 1 urine collection at 2 different times (baseline and at the end of the intervention). BP (baseline and at the end of the intervention).	Changes in sodium excretion were −18.5 mmol/day in the intervention group and −8.7 mmol/day in the control group (p=0.528). No effect on BP.
Yasutake (2018), Japan	78 Adults. Intervention group, *n*=42 (34 women, 57 mean years). Control group, *n*=36 (32 women, 60 mean years).	Parallel randomized trial	4-week intervention. September 2014 to November 2014.	Self-monitoring urinary salt-excretion device	A partition cup (proportional sampling method) was used for the 24 h home urine collection and 1/50 of the total urine volume was collected, 1 urine collection at 2 different times (baseline and at the end of the intervention. BP (baseline and at the end of the intervention).	Salt excretion was lower in the intervention group (-1.2 g/day), but not significant when baseline was corrected. There was no difference between groups. No effect on BP.
Yasutake (2019), Japan	100 Female University students. Intervention group, *n*=51 (21 mean years). Control group, *n*=49 (21 mean years).	Parallel randomized trial	4-week intervention. March 2017 to April 2017 and August 2017 to September 2017.	Self-monitoring urinary salt-excretion device	A partition cup (proportional sampling method) was used for the 24 h home urine collection and 1/50 of the total urine volume was collected, 1 urine collection at 2 different times (baseline and at the end of the intervention. BP (baseline and at the end of the intervention).	The difference in the level of urinary sodium (salt) excretion 24 hours before and after the intervention was −315 mg of sodium (−0 ,8 g/salt) for the intervention group. The adjusted mean difference between groups was -44 mg sodium (-0.1g salt), p=0.074. There was a significant decrease in the control group in SBP and DBP from baseline to the end of the intervention. When these were adjusted by an ANCOVA, the change in SBP was still significant, but the change in DBP was not significant in the control group.

Dorsch (2020), USA	100 Hypertensive. Intervention group, *n*=24 (14 women, 57 mean years). Control group, *n*=26 (16 women, 58 mean years).	Single-center, prospective, open-label randomized controlled trial	8-week intervention. June 2017 to March 2019.	LowSalt4Life, a mobile app. The app sent just-in-time contextual adaptive messages to promote behavior change when a participant entered a supermarket, restaurant or home.	24h urinary Na excretion, 1 urine collection at 2 different times (baseline and at the end of the intervention). BP was measured on a biweekly basis by the participant.	The change in the 24-hour urinary sodium excretion was –637 ±1524 mg in the intervention group and –322 ± 1485 mg in the control group (p=0.47). The systolic blood pressure change from baseline to week 8 in the intervention group was –7.5 mmHg while that in the control group was –0.7 mmHg (p=0.12).
Thatthong (2020), Thailand	50 Hypertensive. Intervention group, *n*=25 (40 mean years). Control group, *n*=25 (42 mean years).	Randomized control trial	8-week intervention.	Nutrition education and sodium reduction key messages from innovative technology (LINE) on their smartphones twice a week.	24h urinary Na excretion, 1 urine collection at 2 different times (baseline and at the end of the intervention). BP (baseline and at the end of the intervention).	Sodium excretion increased in the intervention group (from 2981mg/day to 3489mg/day) and decreased in the control group (from 3106 to 2944) with no statistical significance. Systolic BP level decreased from 125 mmHg to 117 mmHg (p = 0.041) and diastolic BP level decreased from 75 mmHg to 71 mmHg (p = 0.001).

NI – No information; NA – Not applicable; BP – Blood pressure; SBP – Systolic blood pressure; DBP – Diastolic blood pressure; IG – Intervention Group; CG – Control group

Table S2. General characteristics of non-randomized studies without statistically significant salt reduction (p<0.05)

Study author (year), country	Participants	Study design	Enrolment start and end dates; length of participant follow-up	Intervention	Method of assessment	Results
Lofthouse (2016), New Zealand	11 healthy adults (39 mean years)	Pilot study	4-week intervention.	Nutrition education by a nutritionist. It included recommendations to use a salt substitute and training to use a free label reading app for smartphones ("FoodSwitch"). Participants received a booklet and label reading card that were developed by the study nutritionist and were referred to additional resources available on the Internet.	24h urinary Na excretion, 1 urine collection at 2 different times (baseline and after intervention).	The difference in mean urinary excretion was -433 mg/day (2342 ± 1247 mg/day at baseline; 1909 ± 1346 mg/day at the end of the intervention, p= 0.211).
Beer-Borst (2019), Switzerlan d	145 Workers. Intervention group, *n*=128 (50.8% women, 46 mean years). Control group, *n*=13 (38.5% women, 48 mean years).	Nonrandomi zed single-arm trial with calibration arm.	12-month intervention. May 2015 to November 2016.	Educational and environmental intervention. The intervention group received an educational program that included five educational workshops. The catering operation management and employees, catering teams of the companies in the intervention group had five training sessions and food analysis in catering operations.	Salt intakes were estimated using three methods during three-day periods at t0 and t12. A food record checklist, which provided semiquantitative information on food sources of Na (day 1–3), a late afternoon spot urine (day 2) and a 24-hour urine (day 3) collection. BP (baseline)	In the intervention group, salt intake decreased by −0.6 g (from 8.7 g to 8.1 g, p=0.192) or 6.9% over 12 months. The mean daily intake of salt in women was 7 g/day and did not change (0 g (-1.2, 1.1)). Salt intake in men decreased by -1.2 g from 10.4 g to 9.2 g (-2.6, 0.2), but this was not statistically significant.

NI – No information; NA – Not applicable; BP – Blood pressure; SBP – Systolic blood pressure; DBP – Diastolic blood pressure; IG – Intervention Group; CG – Control group
